# OmpA-like proteins of *Porphyromonas gingivalis* contribute to serum resistance and prevent Toll-like receptor 4-mediated host cell activation

**DOI:** 10.1371/journal.pone.0202791

**Published:** 2018-08-28

**Authors:** Megumi Inomata, Toshi Horie, Takeshi Into

**Affiliations:** Department of Oral Microbiology, Division of Oral Infections and Health Sciences, Asahi University School of Dentistry, Hozumi, Mizuho, Gifu, Japan; Universidad Nacional de la Plata, ARGENTINA

## Abstract

*Porphyromonas gingivalis* possesses various abilities to evade and disrupt host immune responses, by which it acts as an important periodontal pathogen. *P*. *gingivalis* produces outer membrane protein A (OmpA)-like proteins (OmpALPs), Pgm6 and Pgm7, as major *O*-linked glycoproteins, but their pathological roles in *P*. *gingivalis* infection are largely unknown. Here, we report that OmpALP-deficient strains of *P*. *gingivalis* show an enhanced stimulatory activity in coculture with host cells. Such an altered ability of the OmpALP-deficient strains was found to be due to their impaired survival in coculture and the release of LPS from dead bacterial cells to stimulate Toll-like receptor 4 (TLR4). Further analyses revealed that the OmpALP-deficient strains were inviable in serum-containing media although they grew normally in the bacterial medium. The wild-type strain was able to grow in 90% normal human serum, while the OmpALP-deficient strains did not survive even at 5%. The OmpALP-deficient strains did not survive in heat-inactivated serum, but they gained the ability to survive and grow in proteinase K-treated serum. Of note, the sensitivity of the OmpALP-deficient strains to the bactericidal activity of human β-defensin 3 was increased as compared with the WT. Thus, this study suggests that OmpALPs Pgm6 and Pgm7 are important for serum resistance of *P*. *gingivalis*. These proteins prevent bacterial cell destruction by serum and innate immune recognition by TLR4; this way, *P*. *gingivalis* may adeptly colonize serum-containing gingival crevicular fluids and subgingival environments.

## Introduction

*Porphyromonas gingivalis*, a gram-negative, anaerobic, and asaccharolytic rod, is a commensal bacterium of subgingival flora [1] and known as one of the most important pathogenic agents of chronic periodontitis [2, 3]. Dissemination of propagated *P*. *gingivalis* from the periodontium lesions causes systemic health problems, such as cardiovascular diseases, aspiration pneumonia, preterm birth, and low birth weight [4–6].

Accumulated evidence has shown that *P*. *gingivalis* possesses a variety of abilities to evade host bactericidal mechanisms and to disarrange innate and adaptive immune responses. For example, trypsin-like cysteine proteases termed gingipains, which are composed of arginine residue-specific gingipains (Rgps) and lysine residue-specific gingipain (Kgp), cleave to activate complement components for inflammatory reactions when *P*. *gingivalis* cells are present in low numbers, whereas gingipains potently degrade complement components to invalidate their bactericidal activity when *P*. *gingivalis* cells grow abundantly [7, 8]. *P*. *gingivalis* also evades Toll-like receptor (TLR)-mediated innate immune responses through production of heterogeneous and atypical forms of lipopolysaccharide (LPS) that have an immunologically weak stimulatory activity toward TLR4 and thereby antagonize TLR4 recognition of other bacterial LPS [9, 10]. In addition, gingipains selectively degrade cell surface CD14, which serves as a coreceptor of TLR4, to hamper induction of TLR4-mediated bactericidal responses [11, 12]. Potent proteolytic properties of gingipains also abrogate the functions of neutrophils, including chemotaxis, oxidative burst, granule-mediated antimicrobial activity, and phagocytic killing [8, 13]. Furthermore, *P*. *gingivalis* affects T-cell functions through suppression of the production of IL-2 and interferon (IFN)-γ to impair T helper 1 responses and disrupts the Th17/regulatory T (Treg) balance via induction of pro-Th17 cytokines and downregulation of TGF-β1 and of the numbers of Treg cells [14]. These abilities ensure survival of *P*. *gingivalis* cells even under highly inflammatory and bactericidal conditions and lead to disturbances of the homeostasis in the periodontium.

Among such abilities against immune responses, the most important and primary one may be the resistance to the bactericidal activity of serum because subgingival *P*. *gingivalis* cells are exposed to gingival crevicular fluids that are derived from serum and contain high concentrations of complement components and bactericidal factors [15, 16]. Serum resistance of *P*. *gingivalis* is thought to be mediated by at least three mechanisms: i) degradation of complement components and immunoglobulins (IgG and IgM) by large-scale production of proteases [7, 17]; ii) binding of the complement inactivator C4b-binding protein (C4BP) to the surface for inactivation of the complement system [18]; and iii) cell surface protection by the synthesis of capsular anionic polysaccharide [19]. However, other mechanisms or components involved in serum resistance have not been clarified.

*P*. *gingivalis* produces outer membrane proteins termed Pgm1–Pgm7 [20]. Pgm6 (also called PG33 or Omp41) and Pgm7 (also called PG32 or Omp40) were found to have C-terminal regions highly similar to the C-terminal peptidoglycan-binding domain of *Escherichia coli* outer membrane protein A (OmpA) [21]. OmpA has an N-terminal domain with an eight-stranded structure that is embedded in the outer membrane and is thought to function as a monomeric porin protein [22]. In contrast, the above-mentioned OmpA-like proteins (OmpALPs) Pgm6 and Pgm7 have only one putative transmembrane region in the N-terminus and are thought to not function as porin proteins [23]. The proteins Pgm6 and Pgm7 form stable heterotrimeric complexes or homotrimers of Pgm7, but homotrimers of Pgm6 are unstable and prone to degradation [23]. These complexes are responsible for the maintenance and stability of the outer membrane. In addition, these OmpALPs are produced as *O*-linked β-*N*-acetylglucosamine (*O*-GlcNAc)-containing glycoproteins and can bind to several kinds of lectins and extracellular matrices in vitro [24]. OmpA of Enterobacteriaceae family bacteria is known to participate in various pathogenic phenomena, including cell adhesion, invasion, or intracellular survival, as well as serum resistance and evasion of killing by complement components [22]. However, it is unknown whether OmpALPs of *P*. *gingivalis*, a Porphyromonadaceae family bacterium, have functions similar to those of OmpA.

In the present study, we aimed to investigate the pathogenic role of the OmpALPs Pgm6 and Pgm7 of *P*. *gingivalis*. In particular, we studied it with interest in examining their ability to interact with host cells by means of the OmpALP-deficient strains of *P*. *gingivalis* in a cultured cell-based infection model. Unexpectedly, these mutant strains were found not to survive in cell culture and to release LPS to stimulate cocultured cells through TLR4. Although these mutant strains normally grew in the bacterial medium equivalently to the parental wild-type (WT) strain, the mutant strains were inviable in serum-containing media. Thus, we provide evidence for a function of Pgm6 and Pgm7 in serum resistance of *P*. *gingivalis* by which *P*. *gingivalis* may prevent TLR4-mediated activation of innate immune responses.

## Materials and methods

### Bacterial strains and growth conditions

*P*. *gingivalis* ATCC 33277 served as a WT strain. The three OmpALP-deficient strains (a Pgm6-deficient strain obtained by deletion of *pg0695* (Δ*695*), a Pgm7-deficient strain obtained by deletion of *pg0694* (Δ*694*), and a Pgm6/7-double deficient strain created via deletion of *pg0695-pg0694* (Δ*695–694*)) constructed from the WT [23] were kind gifts from Dr. Y. Murakami (Asahi University). All the strains were grown in Trypticase soy broth (Becton Dickinson, Franklin Lakes, NJ, USA) supplemented with 2.5 mg/ml yeast extract, 2.5 μg/ml hemin, and 5 μg/ml menadione (sTSB) under anaerobic conditions (10% CO_2_, 10% H_2_, and 80% N_2_). These strains were also anaerobically cultivated in sterile phosphate-buffered saline (PBS) containing 10% of fetal bovine serum (FBS; Thermo Fisher Scientific, Waltham, MA, USA) inactivated by heat treatment at 56°C for 30 min or various concentrations of normal human serum (NHS; Biowest, Nuaillé, France). Bacterial growth was monitored by measuring optical density at 600 nm (OD_600_), and bacterial cell numbers were also estimated by measurement of OD_600_. Enzymatic activities of WT and Δ*695–694* were assessed by means of an API ZYM system (BioMerieux, Marcy-l'Etoile, France) using logarithmic-phase bacteria, according to the manufacturer’s instructions. The activities of Rgp and Kgp in the bacterial culture were determined by measuring OD_405_ for the hydrolysis of the synthetic chromogenic substrates Nα-benzoyl-L-Arg-*p*-nitroanilide and N-*p*-tosyl-Gly-Pro-Lys-*p*-nitroanilide (Sigma-Aldrich, St Louis, MO, USA), respectively [25]. The value of OD_405_ was divided by the value of OD_600_ to obtain the normalized data.

### Detection of bacterial ATP production

Bacterial cultures were prepared in sTSB, Dulbecco’s Modified Eagle’s Medium (DMEM; Wako Pure Chemical Industries, Osaka, Japan) supplemented with 10% of FBS, serum-containing PBS, PBS containing human β-defensin-3 (Peptide Institute, Osaka, Japan) supplemented with 25% of sTSB or PBS containing human lysozyme (Sigma-Aldrich) supplemented with 25% of sTSB. To assess bacterial survival and growth, production of ATP within these bacterial cultures was determined by ATP-driven luminescence using the luciferin substrate and luciferase enzyme of the BacTiter Glo Microbial Cell Viability Assay Kit (Promega, Madison, WI, USA). Bacterial cultures (100 μl) and reagents (100 μl) were mixed in the wells of 96F Nontreated White Microwell SI plates (Thermo Fisher Scientific). Bioluminescence was measured on an Infinite M200 PRO plate reader (Tecan, Seestrasse, Switzerland) and is shown as relative light units (RLUs).

### SYTO9/propidium iodide (PI) staining of bacterial cells

The outer membrane integrity and viability of bacterial cells were assessed by fluorescent staining with the green fluorescent nucleic acid stain SYTO9 and the red fluorescent nucleic acid stain PI of the LIVE/DEAD BacLight Bacterial Viability Kit (Thermo Fisher Scientific) according to the manufacturer’s instructions. These stains differ both in their spectral characteristics and in their ability to penetrate bacterial cells. When mixed in a recommended proportion, SYTO9 produces green fluorescent staining of bacteria with intact cell membranes, PI produces red fluorescent staining of bacteria with damaged membranes, and the background remains virtually nonfluorescent [26]. Double staining was performed by incubating bacterial cells with the mixture of SYTO9 and PI at room temperature for 15 min. Stained bacterial cells were immediately imaged using a BZ-8000 fluorescence microscope (KEYENCE, Osaka, Japan).

### Cell culture

Human gingival fibroblasts (HGFs) previously isolated under the approval by the Ethics Committee of Asahi University [27, 28] were cultured in antibiotic-free DMEM supplemented with 10% of inactivated FBS. Human umbilical vein endothelial cells (HUVECs) were purchased from Lonza (Basel, Switzerland) and prepared as described elsewhere [29] and were cultured in antibiotic-free endothelial cell growth medium 2 (EGM-2; Lonza) containing 10% of inactivated FBS, hydrocortisone, human recombinant fibroblast growth factor, vascular endothelial growth factor, recombinant insulin growth factor 1, ascorbic acid, and human recombinant epidermal growth factor. These cells were used for experiments from passages 4 to 8. RAW264.7 cells were prepared as described previously [30] and maintained in antibiotic-free DMEM supplemented with 10% of inactivated FBS. All the cells were cultivated at 37°C in the humidified atmosphere containing 5% of CO_2_. For stimulation of the mammalian cells with bacterial cells, 10^5^ of mammalian cells were grown in 12-well plates and stimulated with bacterial cell numbers equivalent to a multiplicity of infection of 100:1 (MOI:100) for HGFs and HUVECs or MOI:10 for RAW264.7 cells for 12 h.

### Small interfering RNA (siRNA) and gene silencing

Stealth siRNA targeting *Tlr2* (MSS216272) and *Tlr4* (MSS211922) as well as Stealth RNAi siRNA negative control medium GC duplex#2 (12935112) were purchased from Thermo Fisher Scientific. RAW264.7 cells were washed once with the Opti-MEM I medium (Thermo Fisher Scientific), and then transfection of siRNA was performed with the Lipofectamine RNAi MAX reagent (Thermo Fisher Scientific). After 6 h, culture media were changed to DMEM supplemented with 10% of inactivated FBS, and incubation was continued for additional 12 h.

### Quantitative reverse-transcription polymerase chain reaction (qRT-PCR)

Total RNA was prepared from HGFs, HUVECs, or RAW264.7 cells using a GenElute mammalian total RNA miniprep kit (Sigma-Aldrich). Total RNA (1 μg) was reverse-transcribed using ReverTraAce reverse transcriptase (Toyobo, Otsu, Japan) using both an oligo(dT)_21_ primer and random hexamer primers. qRT-PCR was conducted using SYBR Premix Ex Taq (TaKaRa, Otsu, Japan) on a thermal cycler Dice Real Time System TP800 (TaKaRa). All the predesigned primer sets used for PCR (for genes *IL6*, *IL8*, *GAPDH*, *Il6*, *Cxcl2*, and *Ppia*) were acquired from TaKaRa. The assessment of gene expression was done by the ΔΔ*C*_*t*_ method. Results shown as relative expression of genes of interest were normalized to the levels of the housekeeping gene *GAPDH* in HGFs and HUVECs or *Ppia* in RAW264.7 cells.

### Determination of LPS concentrations

LPS concentrations in bacterial cultures were colorimetrically determined by a Limulus Color KY Test (Wako, Osaka, Japan) and an Infinite M200 PRO plate reader. LPS derived from *P*. *gingivalis* ATCC 33277 acquired from InvivoGen (San Diego, CA, USA) was used as standard samples.

### Statistical analysis

Data expressed as mean ± standard deviation (SD) were subjected to one-way factorial analysis of variance (ANOVA) followed by Dunnett’s pairwise comparison test. A two-tailed *p* value < 0.05 was assumed to denote statistical significance.

## Results

### OmpALP-deficient *P*. *gingivalis* cells grow normally in bacterial culture media

**Fig 1:**
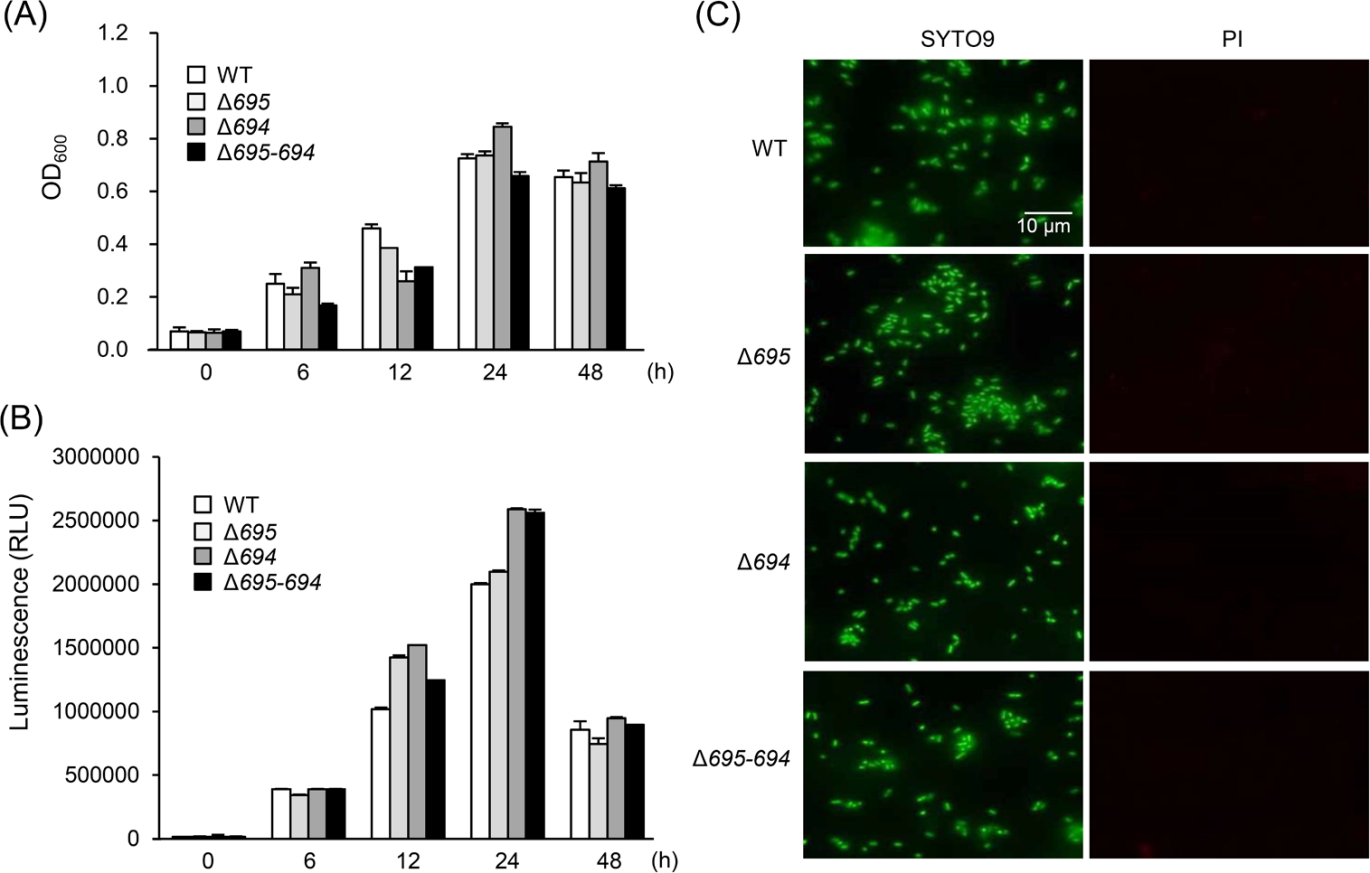
Survival and growth of the OmpALP-deficient strains of *P*. *gingivalis* in sTSB. Bacterial cells (10^7^) of the strains WT, Δ*695*, Δ*694*, and Δ*695–694* were anaerobically cultured in 1 ml of sTSB for the indicated periods. (A and B) The growth was monitored by measuring OD_600_ (A), and the survival was assessed by ATP production (B). Each value is expressed as mean ± SD (n = 3). (C) The integrity of outer membranes was assessed by double fluorescent staining of bacteria (cultured for 12 h) with SYTO9 and PI. Images were captured by means of a fluorescence microscope.

OmpALPs, Pgm6 and Pgm7, are major cell surface proteins of *P*. *gingivalis* [20] and are produced as *O*-GlcNAc-containing glycoproteins [24]. Although these proteins have an ability to bind to several kinds of lectins and extracellular matrices in vitro [24], their specific role in the pathogenicity of *P*. *gingivalis* is largely unclear. To investigate this, we prepared three OmpALP-deficient strains of *P*. *gingivalis*: a Pgm6-deficient strain (Δ*695)*, a Pgm7-deficient strain (Δ*694)*, and a Pgm6/7-double deficient strain (Δ*695–694*) as well as the WT. All the mutant strains as well as the WT were confirmed to normally grow in sTSB, peaking at ~24 h after inoculation (Fig 1A). Additionally, their survival assessed by ATP production in sTSB was almost identical to that of the WT within the time frame of 48 h (Fig 1B). Besides, SYTO9/PI staining of bacterial cells indicated normal integrity of their outer membrane and survival in sTSB (Fig 1C). Furthermore, there was no distinct difference in enzymatic activities, including a trypsin-like protease activity between the WT and Δ*695–694* (S1A Fig). The activities of Rgp and Kgp of the WT were almost identical with those of Δ*695–694* (S1B Fig).

### OmpALP-deficient *P*. *gingivalis* cells are destroyed in cell culture media to stimulate TLR4

**Fig 2:**
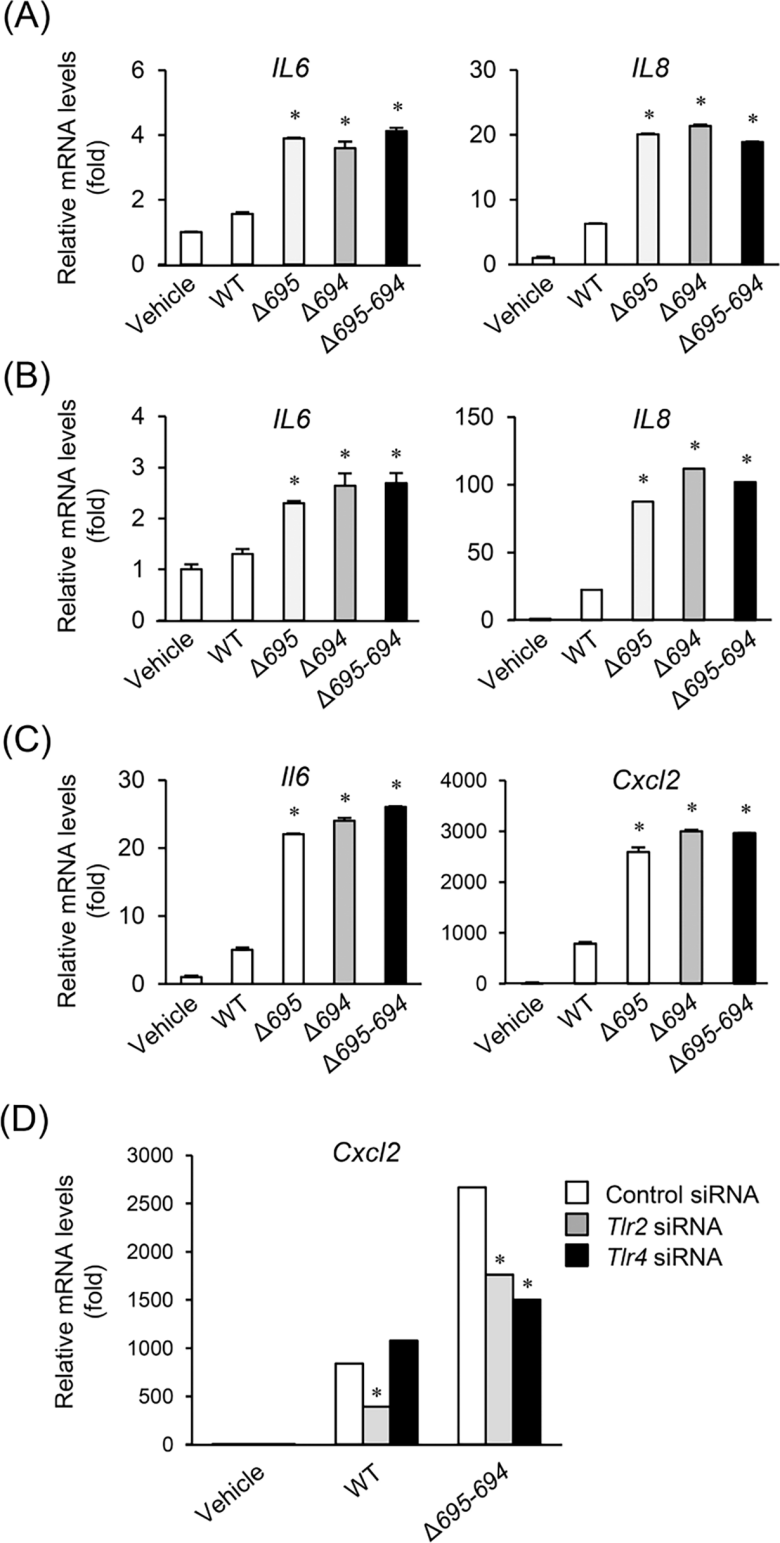
Involvement of TLR2 and TLR4 in the cell-stimulatory activities of the OmpALP-deficient strain of *P*. *gingivalis*. (A-C) HGFs, HUVECs, and RAW264.7 cells (10^5^) were grown in 12-well plates in an antibiotic-free culture medium containing 10% of inactivated FBS. Bacterial cells of WT, Δ*695*, Δ*694*, or Δ*695–694* resuspended in PBS were added to the cell cultures (at MOI: 100 for HGFs and HUVECs; at MOI: 10 for RAW264.7 cells). The cells were incubated for 12 h, followed by RNA extraction. Relative expression levels of *IL6* and *IL8* in HGFs (A) and HUVECs (B) and, *Il6* and *Cxcl2* in RAW264.7 cells (C) were determined by qRT-PCR. Each value, expressed as a fold increase, is mean ± SD (n = 3); *p < 0.05 (compared to vehicle), one-way ANOVA and Dunnett’s test for post hoc comparisons (μc < μi). (D) RAW264.7 cells were transfected with siRNA targeting *Tlr2* or *Tlr4* or with control siRNA. Cells cultured in an antibiotic-free culture medium containing 10% of inactivated FBS were then stimulated with bacterial cells of WT or Δ*695–694* resuspended in PBS at MOI: 10. The cells were incubated for 12 h, followed by RNA extraction. Relative expression levels of *Cxcl2* were determined by qRT-PCR. Each value, expressed as a fold increase, is mean ± SD (n = 3); *p < 0.05 (compared to the Control siRNA), one-way ANOVA and Dunnett’s test for *post hoc* comparisons (μc ≠ μi).

To investigate effects of OmpALP deficiency on the pathogenicity of *P*. *gingivalis* cells against host cells, equivalent numbers of logarithmic-phase bacterial cells were prepared and used in cell-based infection models. As host cells, HGFs, HUVECs, or RAW264.7 macrophage-like cells were used. First, effects of the OmpALP-deficient strains on host cells were investigated by evaluation of the transcription of *IL6* and *IL8* in HGFs infected with bacterial cells at MOI:100. Unexpectedly, all the mutant strains had the upregulated stimulatory activities as compared with the WT (Fig 2A). Increases in the activities of the mutant strains were also observed in the transcription of *IL6* and *IL8* in HUVECs (MOI:100; Fig 2B) and in the transcription of *Il6* and *Cxcl2* in RAW264.7 cells stimulated at MOI:10 (Fig 2C). Some studies have shown that *P*. *gingivalis*-induced host cell activation proceeds through TLR2-mediated recognition of fimbriae [31, 32] and TLR4-mediated recognition of LPS [33, 34]. We therefore tested a gene knockdown of TLR2 and TLR4 using siRNA. In RAW264.7 cells, high knockdown efficiency for *Tlr2* and *Tlr4* at nearly 90% was obtained by siRNA transfection (S2 Fig). The stimulatory ability of the WT to induce transcription of *Cxcl2* was significantly impaired by the *Tlr2* knockdown, whereas the *Tlr4* knockdown hardly affected it (Fig 2D). In contrast, the stimulatory activity of Δ*695–694* was significantly impaired by either knockdown (Fig 2D). Thus, deficiency of OmpALPs causes enhanced TLR4-mediated stimulatory activity on host cells.

**Fig 3:**
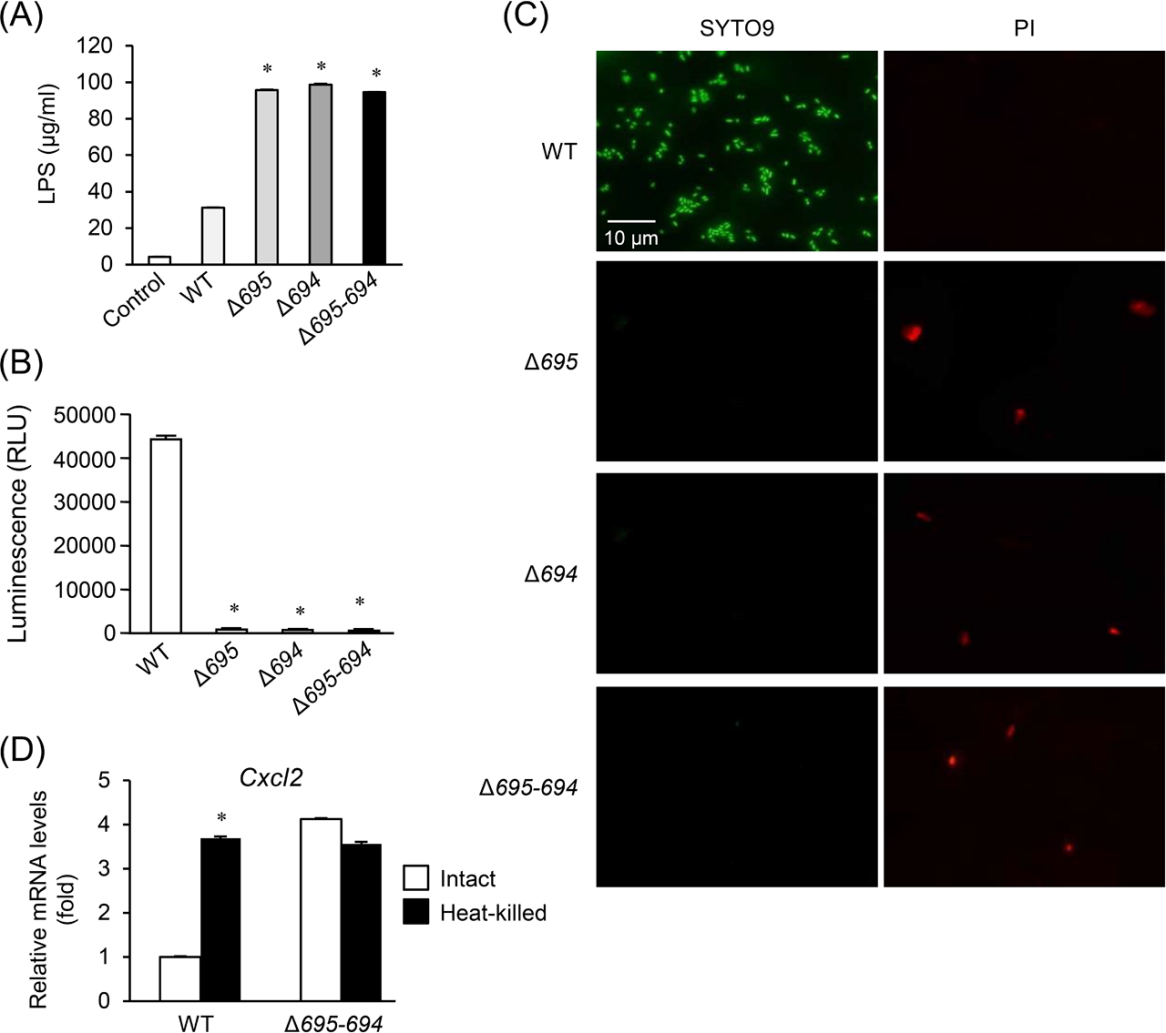
Survival and growth of the OmpALP-deficient strains of *P*. *gingivalis* in DMEM containing 10% of inactivated FBS. (A–C) Bacterial cells (10^7^) of strains WT, Δ*695*, Δ*694*, and Δ*695–694* resuspended in PBS were anaerobically cultured in 1 ml of DMEM containing 10% of inactivated FBS for 6 h. LPS concentrations in the culture supernatants were determined (A). Each value is expressed as the mean ± SD (n = 3); *p < 0.05 (compared to PBS control), one-way ANOVA and Dunnett’s test for *post hoc* comparisons (μc < μi). Their survival was assessed by ATP production (B). Each value is expressed as mean ± SD (n = 3); *p < 0.05 (compared to the WT), one-way ANOVA and Dunnett’s test for *post hoc* comparisons (μc ≠ μi). The integrity of outer membranes was also assessed by fluorescent staining of bacteria (cultured for 12 h) with SYTO9 and PI (C). Images were captured by means of a fluorescence microscope. (D) Bacterial cells (10^7^) of strains WT and Δ*695–694* resuspended in PBS were left untreated (‘Intact’) or boiled for 30 min (‘Heat-killed’). RAW264.7 cells grown in 24-well plates were stimulated with these bacterial cells at MOI 10 for 12 h, followed by RNA extraction. Relative expression levels of *Cxcl2* were determined by qRT-PCR. Each value, expressed as a fold increase, is mean ± SD (n = 3); *p < 0.05 (compared to group Intact), one-way ANOVA and Dunnett’s test for *post hoc* comparisons (μc ≠ μi).

As bacterial fimbriae are exposed to the outer environment, intact *P*. *gingivalis* cells can directly stimulate TLR2 [35]. On the other hand, the lipid A moieties, which are responsible for the core biological activity of LPS, are normally hidden within the outer membrane [36]. Therefore, intact *P*. *gingivalis* cells may be unable to directly stimulate TLR4 unless bacterial cells are injured to expose the lipid A moieties and are lysed to release LPS. We therefore tested whether the deficiency of OmpALPs causes an LPS release into the extracellular environment. Of note, LPS concentrations in the culture supernatants of HGFs stimulated with the OmpALP-deficient strains were significantly higher than those of the WT (Fig 3A). Additionally, bacterial survival in terms of production of ATP was diminished in the OmpALP-deficient strains (Fig 3B). SYTO9/PI staining revealed that intact bacterial cells were almost completely lost, and dead cells were aggregated (Fig 3C). Moreover, in coculture with RAW264.7 cells, although the stimulatory activity of heat-killed WT cells was considerably higher than that of intact WT cells, the stimulatory activity of intact Δ*695–694* cells was similar to that of heat-killed Δ*695–694* cells (Fig 3D). This result indicates that Δ*695–694* cells were dead in the coculture. Thus, deficiency of OmpALPs in *P*. *gingivalis* impairs bacterial survival in the cell coculture, resulting in LPS release and upregulated stimulation of TLR4-mediated responses.

### OmpALP deficiency causes unviability in serum

A previous study showed that the OmpALP-deficient strains can grow in cell-free DMEM supplemented with 1% BSA [23]. On the other hand, these strains could not grow in cell-free DMEM supplemented with 10% of inactivated FBS even under anaerobic conditions (data not shown). We therefore tested the survival and growth of the OmpALP-deficient strains in PBS containing 10% of inactivated FBS. Within the time frame of 48 h, the WT showed unhampered growth, peaking around 24 h after inoculation, whereas the cell numbers of the OmpALP-deficient strains gradually decreased (S3A Fig). In line with this finding, the survival of the OmpALP-deficient strains in terms of production of ATP gradually decreased and disappeared at 48 h after inoculation (S3B Fig). Thus, inactivated FBS causes death of OmpALP-deficient *P*. *gingivalis*.

**Fig 4:**
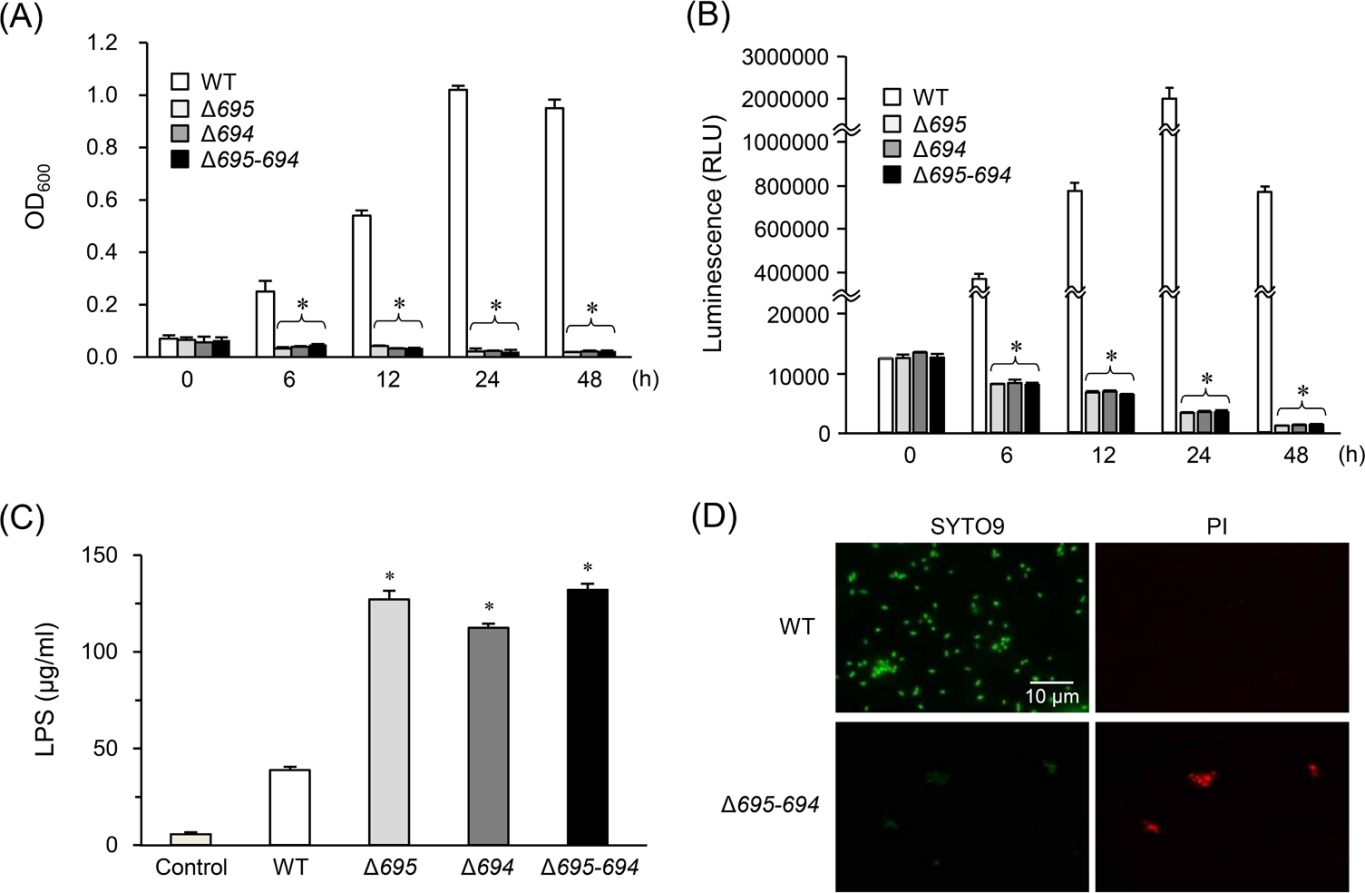
Survival and growth of the OmpALP-deficient strains of *P*. *gingivalis* in 10% NHS. Bacterial cells (10^7^) of strains WT, Δ*695*, Δ*694*, and Δ*695–694* resuspended in 0.9 ml of PBS were mixed with 0.1 ml of NHS and anaerobically cultured for the indicated periods. (A and B) Their growth was monitored by measuring OD_600_ (A), and their survival was assessed by ATP production (B). Each value is expressed as mean ± SD (n = 3); *p < 0.05 (compared to the WT), one-way ANOVA and Dunnett’s test for *post hoc* comparisons (μc ≠ μi). (C) LPS concentrations in the supernatants of these cultures (6 h) were determined (A). Each value is expressed as mean ± SD (n = 3); *p < 0.05 (compared to the Control), one-way ANOVA and Dunnett’s test for *post hoc* comparisons (μc < μi). (D) The integrity of outer membranes was also assessed by fluorescent staining of bacteria (cultured for 12 h) with SYTO9 and PI. The images were acquired by means of a fluorescence microscope. The results on strains Δ*695* and Δ*694* are shown in S5 Fig.

We next investigated survival and growth of the OmpALP-deficient strains in PBS containing 10% NHS. Similarly to the results on inactivated FBS, the WT strain could grow in PBS containing 10% NHS, peaking at 24 h after inoculation, but the OmpALP-deficient strains could not grow (Fig 4A). Similar results were observed on their survival assessed by ATP production (Fig 4B). Survival of the WT and Δ*695–694* in PBS during a 6-h period was almost same (S4 Fig). In PBS containing 10% NHS, however, the WT strain was able to grow prominently, whilst the levels of survival of OmpALP-deficient strains were declined similarly to the levels observed in PBS (Fig 4B). Furthermore, LPS concentrations in the supernatants of OmpALP-deficient strains were significantly higher than those of WT cells at 12 h after inoculation (Fig 4C). SYTO9/PI staining revealed that the OmpALP-deficient bacterial cells were dead and aggregated in contrast to the surviving WT cells (Fig 4D and S5 Fig).

**Fig 5:**
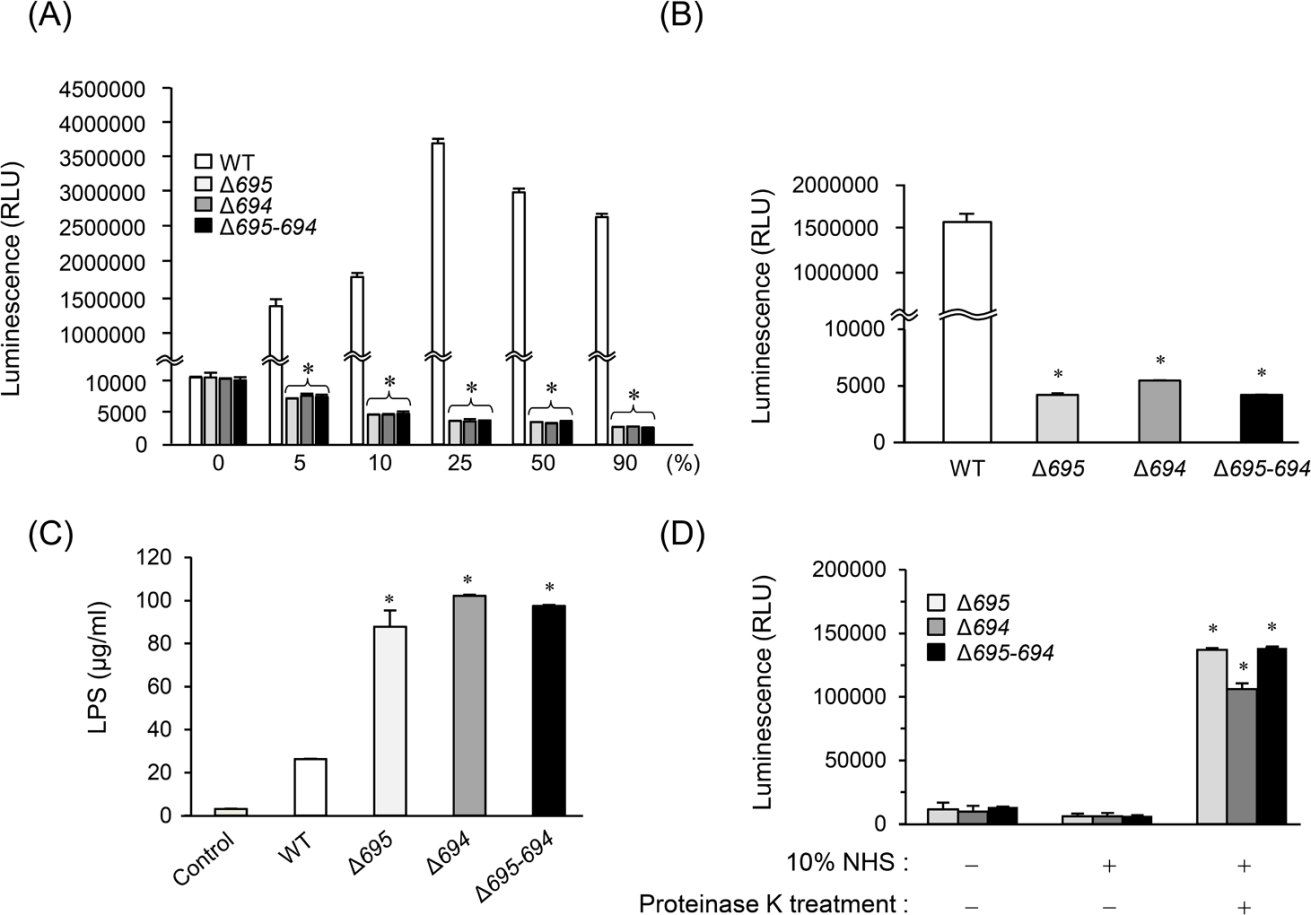
Survival and growth of the OmpALP-deficient strains of *P*. *gingivalis* in NHS. (A) Bacterial cells (10^7^) of strains WT, Δ*695*, Δ*694*, and Δ*695–694* resuspended in 0.1 ml of PBS were mixed with 0.9 ml of indicated concentrations of diluted NHS and anaerobically cultured for 24 h. Bacterial survival was assessed by ATP production. Each value is expressed as mean ± SD (n = 3); *p < 0.05 (compared to the WT), one-way ANOVA and Dunnett’s test for *post hoc* comparisons (μc ≠ μi). (B and C) Effects of heat-inactivation of NHS. Bacterial cells (10^7^) of strains WT, Δ*695*, Δ*694*, and Δ*695–694* were cultured in 10% heat-inactivated NHS for 24 h. Bacterial survival was assessed by ATP production (B). LPS concentrations in the supernatants of the cultures (6 h) were determined (C). Each value is expressed as mean ± SD (n = 3); *p < 0.05 (compared to the Control), one-way ANOVA and Dunnett’s test for *post hoc* comparisons (μc ≠ μi). (D) Effects of proteinase K treatment of NHS. Bacterial cells (10^7^) of strains Δ*695*, Δ*694*, and Δ*695–694* were cultured in 10% proteinase K-treated or untreated NHS for 24 h. Bacterial survival was assessed by ATP production. Each value is expressed as mean ± SD (n = 3); *p < 0.05, one-way ANOVA and multiple Dunnett’s test for *post hoc* comparisons between the groups of interest (μc ≠ μi).

We further assessed the details of survival and growth of the OmpALP-deficient strains in NHS. At 24 h after inoculation, the WT was able to survive in PBS and able to grow in PBS containing NHS ranging from 5% to 90% (peaking at 25%; Fig 5A). In contrast, although the OmpALP-deficient strains were able to survive in PBS, they could not survive in PBS containing NHS even at 5% (Fig 5A). The picture of the bacterial cultures in PBS containing 50% NHS clearly showed that the WT grew normally with production of black sediments, while Δ*695–694* could not grow (S6 Fig). Heat treatment (56°C, 30 min) of NHS for inactivation of complement components, which abrogates all pathways of complement activation [37, 38], did not affect the survival of the OmpALP-deficient strains (Fig 5B). These strains were disrupted and released LPS even during cultivation with inactivated NHS (Fig 5C). We additionally tested proteinase K treatment of NHS to completely destroy the functions of serum proteins, including bactericidal proteins, and found that OmpALP-deficient strains gained the ability to survive and grow only in the treated serum (Fig 5D). These results collectively suggest that heat-insensitive serum protein(s) possess a bactericidal activity against the OmpALP-deficient strains; this activity can be neutralized by the WT.

### OmpALP-deficient *P*. *gingivalis* cells are sensitive to β-defensin 3 (hBD-3)

**Fig 6:**
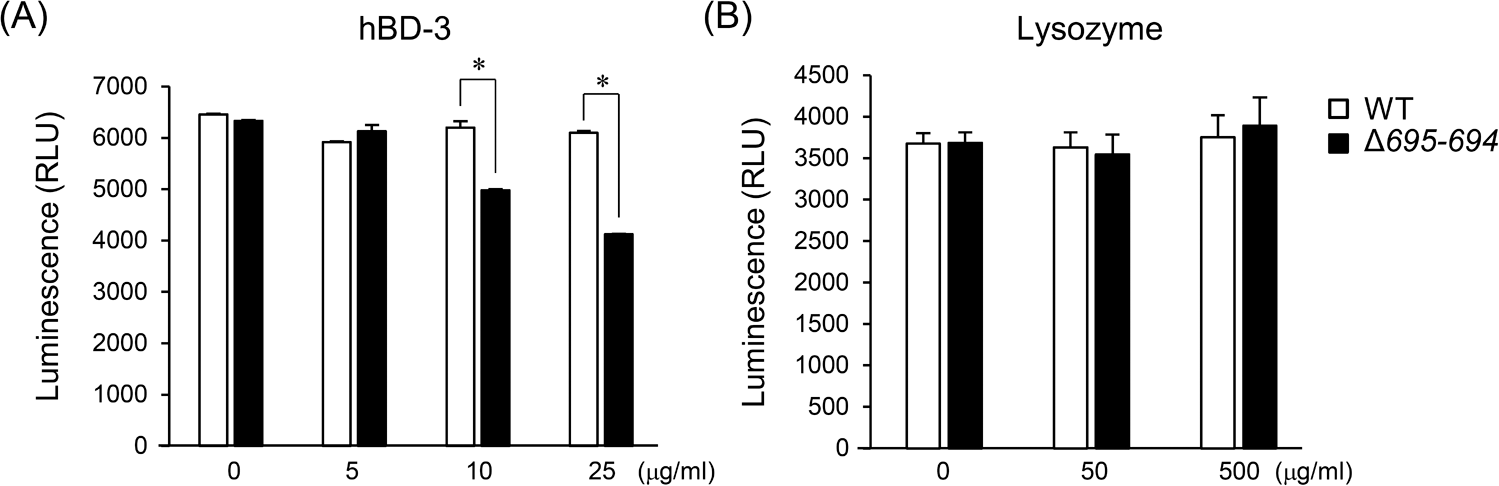
The sensitivity of the OmpALP-deficient strains of *P*. *gingivalis* to the bactericidal activities of hBD-3 and lysozyme. (A and B) Bacterial cells (10^7^) of strains WT and Δ695–694, resuspended in 0.25 ml sTSB, were mixed with 0.75 ml of the indicated concentrations of diluted anti-microbial agents and anaerobically cultured for 6 h. Bacterial survival was assessed by ATP production. Each value is expressed as mean ± SD (n = 3); *p < 0.05, one-way ANOVA and Dunnett’s test for post hoc comparisons (μc ≠ μi).

Our observations suggest that the killing of OmpALP-deficient strains can be mediated by serum proteins whose activity is not abrogated by heat treatment. Low-molecular-weight bactericidal peptides, such as defensins, may be such candidates. Among of them, hBD-3 was previously reported to suppress the growth of *P*. *gingivalis* [39], though its activity can be suppressed by gingipains [40]. We tested the sensitivity of the OmpALP-deficient strains to hBD-3, and found it to be increased as compared with the WT (Fig 6A). However, sensitivity of the OmpALP-deficient strains to lysozyme, a cell wall-degrading enzyme, was identical to that of the WT (Fig 6B), suggesting that the stability of the outer membrane of OmpALP-deficient strains is not altered by OmpALP deficiency.

## Discussion

This study was aimed at investigating a previously unknown role of the OmpALPs Pgm6 and Pgm7 in *P*. *gingivalis* pathogenicity. Experiments were carried out using OmpALP-deficient strains by comparing their properties to those of the WT. We found that the OmpALP-deficient strains had considerably increased abilities to stimulate TLR4-mediated host cell responses. This phenomenon was eventually found to be caused by the destruction of the OmpALP-deficient strains to release LPS in serum-containing media, as they possessed lower resistance to the bactericidal activity of the serum. The results of heat treatment and proteinase K treatment of NHS indicated that protein components with heat-insensitive properties are involved in the killing of the OmpALP-deficient strains. Thus, OmpALPs protect the bacterial cell surface from bactericidal attacks by serum components, such as hBD-3, thereby allowing *P*. *gingivalis* to prevent LPS exposure as well as evasion of TLR4 recognition and elimination by innate immunity. Our findings highlight a previously unknown function of OmpALPs in the pathogenicity of *P*. *gingivalis*.

Gingipains consist of three proteases encoded by different genes: *rgpA*-derived three different isoforms, HRgpA, RgpA, and mt-RgpA; *rgpb*-derived RgpB; and *kgp*-derived Kgp [41]. These trypsin-like proteases are responsible for 85% of the general proteolytic activity of *P*. *gingivalis* [42]. Gingipains degrade a wide range of host proteins, including proteins for immune protection against infections; therefore, they are thought to be important etiological agents for serum resistance of *P*. *gingivalis* [7, 18]. Actually, gingipains degrade complement components C3, C4, and C5 [7]. Moreover, a *P*. *gingivalis* mutant lacking gingipains shows increased deposition of complement components on the cell surface [7, 19] and has considerably higher susceptibility to killing by complement [43]. A previous study has shown that the OmpALP-deficient strains can produce normal or rather upregulated quantity of gingipains [25]. Indeed, a trypsin-like protease activity and activities of Rgp and Kgp could be normally found in Δ*695–694* as well as in the WT (S1 Fig). It is therefore possible that the impaired serum resistance of the OmpALP-deficient strains does not involve gingipain-mediated degradation of complement components. This idea is supported by the result indicating that inactivation of serum does not affect the killing of the OmpALP-deficient strains (Fig 5B and 5C). This result is also strongly supported by findings (elsewhere) that gingipain-deficient *P*. *gingivalis* strains still show strong resistance to serum and to complement killing [19]. In contrast, the OmpALP-deficient strains became viable after proteinase K treatment of serum (Fig 5D). This observation suggests that killing of the OmpALP-deficient strains can be mediated by serum components whose activity is not abrogated at least by heat treatment. Low-molecular-weight bactericidal proteins or peptides were thought as such candidates, but some of them, including α-defensin 1; β-defensins 1, 3, and 4A; LL-37; and SLPI, were reported to be inactivated by gingipains [40,44,45]. Our results indicated that hBD-3 is one of the candidates, as the sensitivity of the OmpALP-deficient strains to the bactericidal activity of hBD-3 was significantly higher than that of the WT (Fig 6).

A previous report demonstrated that the mechanisms of capsular polysaccharide synthesis, including PorR and WpbB, are required for serum resistance of *P*. *gingivalis* by means of mutant strains deficient in polysaccharide synthesis [19]. On the other hand, because *P*. *gingivalis* ATCC 33277 is a nonencapsulated strain, the presence of capsular polysaccharide may not be involved in serum resistance of this strain. OmpALPs of *P*. *gingivalis* are present as major *O*-linked glycoproteins that have sugar chains consisting of *O*-linked GlcNAc, mannose, and *N*-acetyl-D-galactosamine [24]. The sugar chains linked to OmpALPs may be directly associated with serum resistance of the strain. In addition, it is possible that the stability of the outer membrane affects serum resistance because OmpALPs are suggested to control the stability of the outer membrane [23]. Exploration of these possibilities will be a crucial task for our future study.

OmpA is a class of surface-exposed porin proteins that are highly conserved among bacteria of the Enterobacteriaceae family and are produced in large amounts [22]. This protein has an N-terminal domain with an eight-stranded structure, which is embedded in the outer membrane, and a C-terminal domain with a globular structure, which is located in the periplasmic space [22]. The C-terminal domain can noncovalently associate with peptidoglycan, a network of glycan chains composed of disaccharides. On the other hand, OmpALPs are a class of proteins containing a C-terminal OmpA-like domain that is homologous to the C-terminal domain of OmpA [21], and OmpALPs are thought to not function as porin proteins [23]. OmpALPs were found to be conserved in Porphyromonadaceae family bacteria, including human commensals, *P*. *endodontalis* and *P*. *asaccharolytica* (our unpublished observations), and in a periodontopathic species, *Tannerella forsythia* [46]. OmpA has been suggested to play pathogenic roles in adhesion, invasion, or intracellular survival as well as serum resistance and evasion of host defense mechanisms, including killing by complement [22]. Moreover, the function of OmpA of *Klebsiella pneumoniae* is similar to that of OmpALPs in *P*. *gingivalis* found in this study. An OmpA-deficient strain of *K*. *pneumoniae* shows increased stimulatory activities in airway cell responses through activation of TLR2-, TLR4-, and NOD1-mediated recognition; accordingly, this mutant is completely excluded from the lung in an animal infection model [47]. Therefore, OmpA and OmpALPs may share similar biological functions to contribute to serum resistance and to evasion of innate immune recognition. It will be necessary to determine whether other periodontopathic pathogens possess such OmpALP-mediated properties.

In this study, three OmpALP-deficient strains (a Pgm6-deficient strain, a Pgm7-deficient strain, and a Pgm6/7-double deficient strain) were used to compare their properties with those of the WT. The OmpALP proteins Pgm6 and Pgm7 were previously reported to form a Pgm6/7 heterotrimer or a homotrimer of Pgm7, though homotrimers of Pgm6 are unstable and prone to degradation [23]. Therefore, these two trimers are thought to be responsible for the maintenance and stability of the outer membrane. In this study, our results clearly show that both single mutant strains have similar properties to those of the double mutant strain. This strongly implies that the Pgm6/7 heterotrimer, but not the Pgm7 homotrimer, is fully responsible for serum resistance. Although a previous study by Nagano et al [23] could not functionality characterize either the heterotrimer and homotrimer, our results indicate that the heterotrimer cannot be replaced by the Pgm7 homotrimer, at least with regard to the serum resistance of *P*. *gingivalis*.

We can conclude from our results that OmpALPs have a previously unknown ability to facilitate serum resistance of *P*. *gingivalis* cells. Our result suggests that the sensitivity of OmpALP-deficient strains to killing by serum is considerably higher than that of the WT. Therefore, it is easy to imagine that OmpALPs play a considerably important role in evasion of immune killing by serum or other body fluids. However, because we used *P*. *gingivalis* ATCC 33277 as a parental strain, there is room for further research on other strains and their mutants deficient in OmpALPs. Further studies will have to address the detailed mechanisms of OmpALP-mediated serum resistance. It should also be determined which serum components kill OmpALP-deficient *P*. *gingivalis*.

## Supporting information

S1 Fig Enzymatic activities, Rgp activity, and Kgp activity of the WT and Δ*695–694* strains of *P*. *gingivalis*. (A) Cultures of *P*. *gingivalis* strains WT and Δ*695–694* in the logarithmic phase of growth (12 h after inoculation) in sTSB were harvested and washed, and the bacterial suspensions were used to assess the activities of the shown enzymes using the API ZYM system.
(B) The cultures of *P*. *gingivalis* strains WT and Δ*695–694* in sTSB were used to assess Rgp and Kgp activities. Each result is expressed as mean ± SD (n = 3).
(PDF)

S2 Fig Knockdown efficiency of siRNAs in RAW264.7 cells. The expression levels of *Tlr2* and *Tlr4* in RAW264.7 cells transfected with siRNA targeting *Tlr2* or *Tlr4*, or with control siRNA, were determined by qRT-PCR to evaluate the knockdown efficiency and specificity of siRNAs. Each value, expressed as a fold increase of relative mRNA levels, is mean ± SD (n = 3); *p < 0.05 (compared to the Control siRNA), one-way ANOVA and Dunnett’s test for *post hoc* comparisons (μc ≠ μi).
(PDF)

S3 Fig Survival and growth of OmpALP-deficient strains of *P*. *gingivalis* in PBS containing 10% of inactivated FBS. Bacterial cells (10^7^) of strains WT, Δ*695*, Δ*694*, and Δ*695–694* were anaerobically cultured in 1 ml of PBS containing 10% of inactivated FBS for the indicated periods.
(A and B) The growth was monitored by measuring OD_600_ (A), and the survival was assessed by ATP production (B). Each value is expressed as mean ± SD (n = 3); *p < 0.05 (compared to the WT), one-way ANOVA and Dunnett’s test for *post hoc* comparisons (μc ≠ μi).
(PDF)

S4 Fig Survival of WT and OmpALP-deficient strains of *P*. *gingivalis* in PBS. Bacterial cells (10^7^) of the strains WT and Δ*695–694* suspended in 1 ml of PBS were anaerobically cultured for the indicated periods. The survival was assessed by ATP production. Each value is expressed as mean ± SD (n = 3).
(PDF)

S5 Fig The integrity of outer membranes in OmpALP-deficient strains of *P*. *gingivalis* in 10% NHS. Bacterial cells (10^7^) of strains WT, Δ*695*, Δ*694*, and Δ*695–694* resuspended in 0.9 ml of PBS were mixed with 0.1 ml of NHS and anaerobically cultured for 12 h. The integrity of outer membranes was assessed by fluorescent staining of bacteria (cultured for 12 h) with SYTO9 and PI. Images were captured by means of a fluorescence microscope. The results on WT and Δ*695–694* are shown in Fig 6.
(PDF)

S6 Fig A picture of the cultures of strains WT and Δ*695–694* grown in 50% NHS. Cultures of WT and Δ*695–694* in the logarithmic phase of growth (at 12 h after inoculation) in sTSB were harvested and washed, and then 0.1 ml of bacterial suspensions in PBS (containing 10^7^ cells) was mixed with 0.5 ml of human serum and 0.4 ml of PBS in test tubes. The mixtures were anaerobically incubated at 37°C for 12 h. The bacterial cultures in test tubes were transferred to the wells of a 24-well plate to take a picture.
(PDF)
