# Supplementary figures and images for: OmpA-like proteins of Porphyromonas gingivalis contribute to serum resistance and prevent Toll-like receptor 4-mediated host cell activation
Source: PLoS One. 2018 Aug 28;13(8):e0202791. doi: 10.1371/journal.pone.0202791 (PMC6112661; doi:10.1371/journal.pone.0202791)

S1 Fig.

(A)

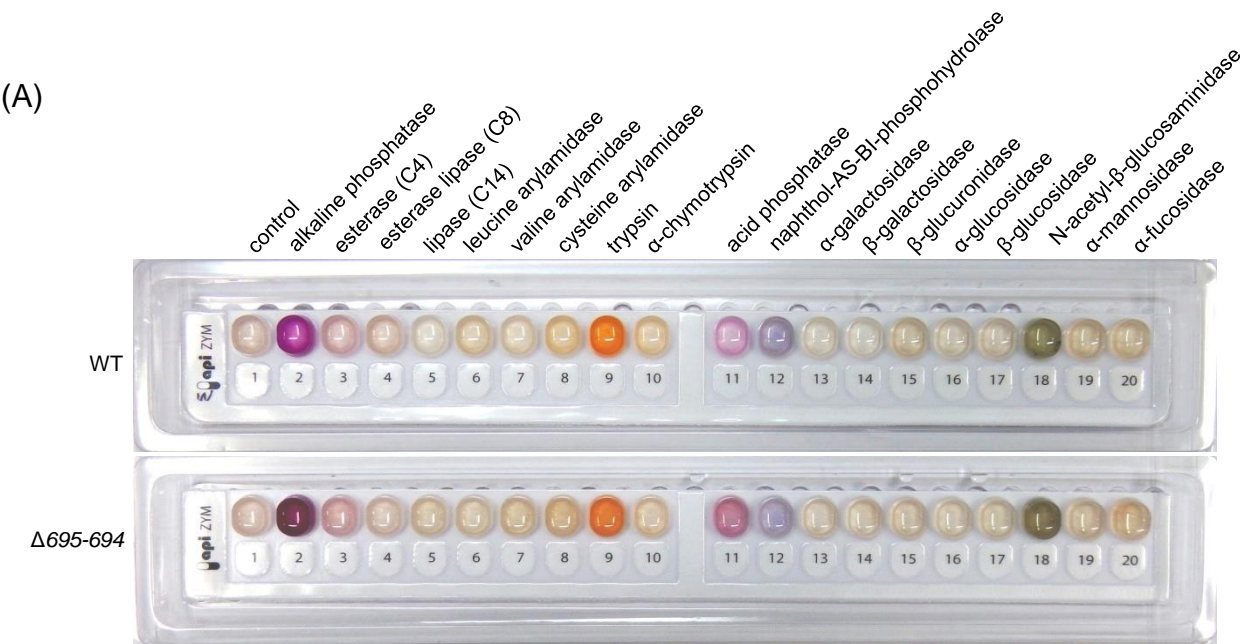

(B)

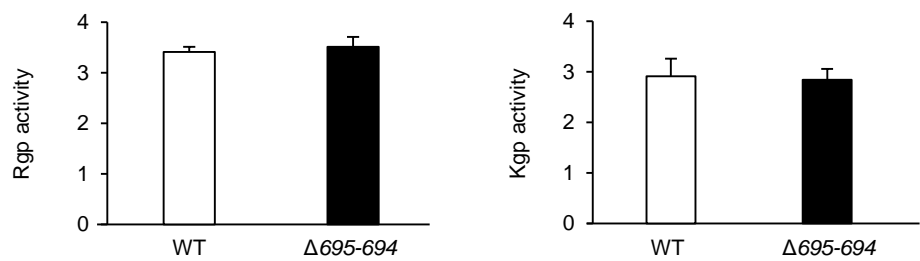

Supplement: S1 Fig — (A) Cultures of P. gingivalis strains WT and Δ695–694 in the logarithmic phase of growth (12 h after inoculation) in sTSB were harvested and washed, and the bacterial suspensions were used to assess the activities of the shown enzymes using the API ZYM system. (B) The cultures of P. gingivalis strains WT and Δ695–694 in sTSB were used to assess Rgp and Kgp activities. Each result is expressed as mean ± SD (n = 3). (PDF) [file pone.0202791.s001.pdf]

S2 Fig.

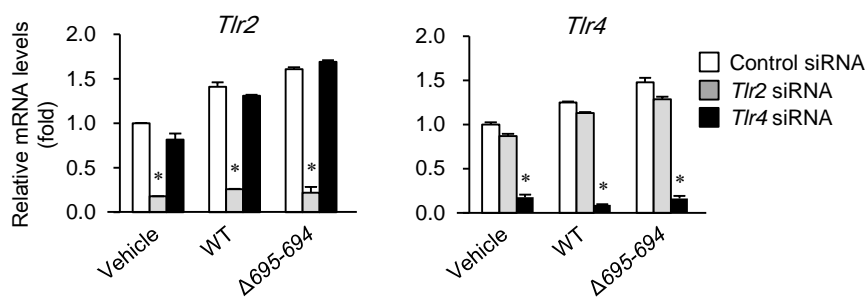

Supplement: S2 Fig — The expression levels of Tlr2 and Tlr4 in RAW264.7 cells transfected with siRNA targeting Tlr2 or Tlr4, or with control siRNA, were determined by qRT-PCR to evaluate the knockdown efficiency and specificity of siRNAs. Each value, expressed as a fold increase of relative mRNA levels, is mean ± SD (n = 3); *p < 0.05 (compared to the Control siRNA), one-way ANOVA and Dunnett’s test for post hoc comparisons (μc ≠ μi). (PDF) [file pone.0202791.s002.pdf]

S3 Fig.

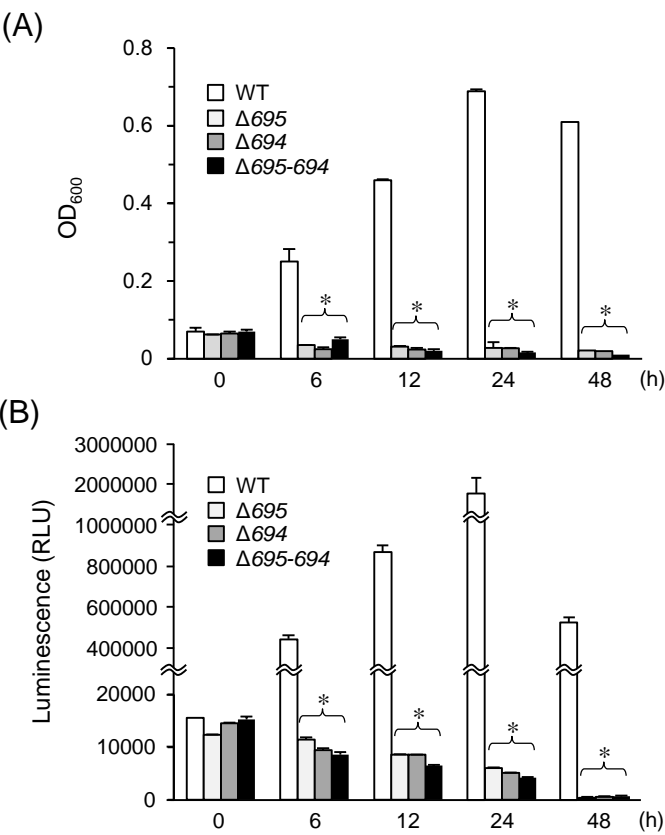

Supplement: S3 Fig — Bacterial cells (107) of strains WT, Δ695, Δ694, and Δ695–694 were anaerobically cultured in 1 ml of PBS containing 10% of inactivated FBS for the indicated periods. (A and B) The growth was monitored by measuring OD600 (A), and the survival was assessed by ATP production (B). Each value is expressed as mean ± SD (n = 3); *p < 0.05 (compared to the WT), one-way ANOVA and Dunnett’s test for post hoc comparisons (μc ≠ μi). (PDF) [file pone.0202791.s003.pdf]

S4 Fig.

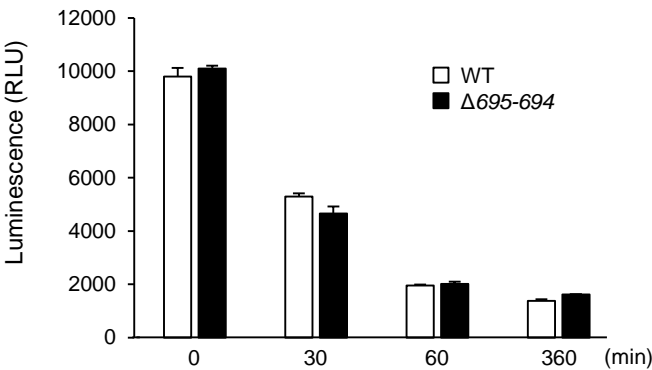

Supplement: S4 Fig — Bacterial cells (107) of the strains WT and Δ695–694 suspended in 1 ml of PBS were anaerobically cultured for the indicated periods. The survival was assessed by ATP production. Each value is expressed as mean ± SD (n = 3). (PDF) [file pone.0202791.s004.pdf]

S5 Fig.

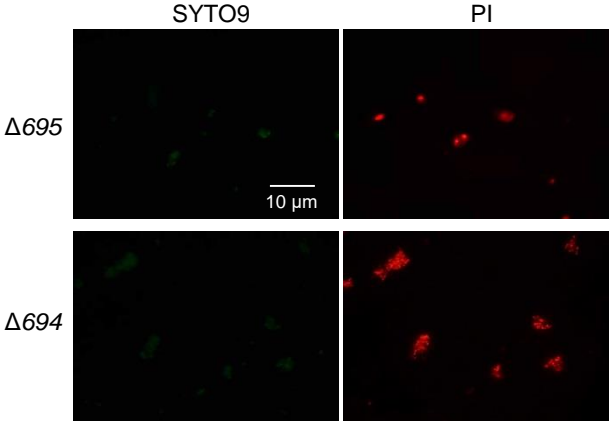

Supplement: S5 Fig — Bacterial cells (107) of strains WT, Δ695, Δ694, and Δ695–694 resuspended in 0.9 ml of PBS were mixed with 0.1 ml of NHS and anaerobically cultured for 12 h. The integrity of outer membranes was assessed by fluorescent staining of bacteria (cultured for 12 h) with SYTO9 and PI. Images were captured by means of a fluorescence microscope. The results on WT and Δ695–694 are shown in Fig 6. (PDF) [file pone.0202791.s005.pdf]

S6 Fig.

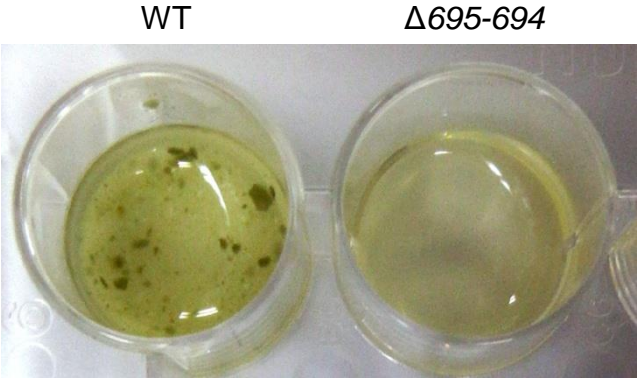

Supplement: S6 Fig — Cultures of WT and Δ695–694 in the logarithmic phase of growth (at 12 h after inoculation) in sTSB were harvested and washed, and then 0.1 ml of bacterial suspensions in PBS (containing 107 cells) was mixed with 0.5 ml of human serum and 0.4 ml of PBS in test tubes. The mixtures were anaerobically incubated at 37°C for 12 h. The bacterial cultures in test tubes were transferred to the wells of a 24-well plate to take a picture. (PDF) [file pone.0202791.s006.pdf]
